# Transcriptome Analysis of *Pennisetum glaucum* (L.) R. Br. Provides Insight Into Heat Stress Responses

**DOI:** 10.3389/fgene.2022.884106

**Published:** 2022-06-02

**Authors:** Albert Maibam, Showkat Ahmad Lone, Sunil Ningombam, Kishor Gaikwad, S. V. Amitha Mithra, Madan Pal Singh, Sumer Pal Singh, Monika Dalal, Jasdeep Chatrath Padaria

**Affiliations:** ^1^ PG School, Indian Council of Agricultural Research-Indian Agricultural Research Institute, New Delhi, India; ^2^ Indian Council of Agricultural Research -National Institute for Plant Biotechnology, New Delhi, India; ^3^ Division of Plant Physiology, Indian Council of Agricultural Research -Indian Agricultural Research Institute, New Delhi, India; ^4^ Division of Genetics, Indian Council of Agricultural Research-Indian Agricultural Research Institute, New Delhi, India

**Keywords:** *Pennisetum glaucum* (L.) R. Br., heat stress (HS), flag leaf, RNA sequencing (RNAseq), SSRs (simple sequence repeats), SNPs (single-nucleotide polymorphisms)

## Abstract

*Pennisetum glaucum* (L.) R. Br., being widely grown in dry and hot weather, frequently encounters heat stress at various stages of growth. The crop, due to its inherent capacity, efficiently overcomes such stress during vegetative stages. However, the same is not always the case with the terminal (flowering through grain filling) stages of growth, where recovery from stress is more challenging. However, certain pearl millet genotypes such as 841-B are known to overcome heat stress even at the terminal growth stages. Therefore, we performed RNA sequencing of two contrasting genotypes of pearl millet (841-B and PPMI-69) subjected to heat stress (42°C for 6 h) at flowering stages. Over 274 million high quality reads with an average length of 150 nt were generated, which were assembled into 47,310 unigenes having an average length of 1,254 nucleotides, N50 length of 1853 nucleotides, and GC content of 53.11%. Blastx resulted in the annotation of 35,628 unigenes, and functional classification showed 15,950 unigenes designated to 51 Gene Ontology terms. A total of 13,786 unigenes were allocated to 23 Clusters of Orthologous Groups, and 4,255 unigenes were distributed to 132 functional Kyoto Encyclopedia of Genes and Genomes database pathways. A total of 12,976 simple sequence repeats and 305,759 SNPs were identified in the transcriptome data. Out of 2,301 differentially expressed genes, 10 potential candidate genes were selected based on log2 fold change and adjusted *p* value parameters for their differential gene expression by qRT-PCR. We were able to identify differentially expressed genes unique to either of the two genotypes, and also, some DEGs common to both the genotypes were enriched. The differential expression patterns suggested that 841-B 6 h has better ability to maintain homeostasis during heat stress as compared to PPMI-69 6 h. The sequencing data generated in this study, like the SSRs and SNPs, shall serve as an important resource for the development of genetic markers, and the differentially expressed heat responsive genes shall be used for the development of transgenic crops.

## Introduction

Temperature is a key physical parameter which affects the growth and development of a plant. According to the Intergovernmental Panel on Climate Change (IPCC), there has been an average increase of 4°C in global atmospheric temperature since the late 20th century (Porter et al., 2014). A plant undergoes a number of morphological, physiological, biochemical, and molecular changes during heat stress to ensure its survival (Wahid et al., 2007). These changes include reduction in chlorophyll content, changes in membrane fluidity and protein stability, production of reactive oxygen species (ROS), secondary signaling, and transcriptional changes. In some crops, the occurrence of heat stress during the flowering period leads to poor grain setting (Iqbal et al., 2009; Moriondo et al., 2011). Increased water stress due to heat throughout the growing cycle can reduce the crop yield (Lobell et al., 2013). Despite the impact of high temperature on plant growth and crop yield being quite profound, the underlying heat tolerance mechanisms are not clearly understood in many crops.

Pearl millet [*Pennisetum glaucum* (L.) R. Br.], also known as *Cenchrus americanus* (L.) Morrone, is widely grown in the African and Indian subcontinents, since prehistoric times. The crop’s main center of diversity is known to be the Sahel zone of West Africa. Pearl millet is a C4 species having diploid number 2n = 14 and genome size around 1.79 Gb (Varshney et al., 2017) and is mostly grown under drought-prone semi-arid and arid tropics in the regions with 200–800 mm of annual rainfall. Pearl millet is grown in an area of about 31 million hectares worldwide in more than 30 countries with more than 90 million poor people depending on it for food and income (http://exploreit.icrisat.org/profile/Pearl%20Millet/178 accessed on 6 February 2022). Optimum temperature required for the growth of pearl millet is about 30–35°C (Maibam and Padaria, 2015). It is well known for its tolerance to extreme environmental conditions. Limited genomic resources are available as compared to other crop species.

Next-generation sequencing (NGS) based technology for the analysis of transcriptome is more powerful and accurate compared to the Sanger based EST sequencing, suppression subtractive hybridization, and hybridization based microarrays (Marioni et al., 2008; Agarwal et al., 2010). Moreover, over the last decade, several NGS platforms including the illumina are becoming more affordable and efficient for transcriptome sequencing. Additionally, transcriptome sequencing has emerged as an alternative core technology for the discovery and understanding of genes associated with desired traits, where full genome sequencing is not economically feasible especially in case of nonmodel plants. RNA-sequencing (RNA-Seq) has become a benchmark tool for whole transcriptome gene expression quantification and identification of differentially expressed genes (DEGs). It provides scope for the identification of probable candidate genes involved in abiotic and biotic stress tolerance and further for the development of molecular markers (Wang et al., 2009; Garber et al., 2011; Strickler et al., 2012). Third generation sequencing technology such as PacBio provides long/full length transcripts but still having high single error base rate (Sun et al., 2020).

Enormous amounts of ESTs generated from various transcriptomic studies and other genomic sequences are available in public databases for many model plant species (James et al., 2015). However, limited research emphasis has been given to the nonmodel crops including pearl millet, as evidenced by the presence of only 75,499 ESTs (https://www.ncbi.nlm.nih.gov/nuccore/?term=pearl+millet+ESTs, Accessed on 12th April 2022) of this crop in GenBank. It is now possible to assemble transcripts without the reference genome *via de novo* assembly using trinity (Haas et al., 2014) and/or one of the several other available software tools. Recently, genome wide expression profiling in various nonmodel plant species growing under abiotic or biotic stresses, for various biosynthetic pathways or developmental stages, has been carried out using RNA-sequencing. These plants include *Raphanus sativus* L. (Nie et al., 2016), *Scrophularia ningpoensis* Hemsl. (Pan et al., 2017), *Camellia sinensis* (L.) Kuntze (Guo et al., 2017), *Lilium* genotypes (Hu et al., 2017), *Brassica rapa* L. (Greenham et al., 2017), *Sesamum indicum* L. (Dossa et al., 2017), *Asparagus officinalis* L. (Li et al., 2017), and *Agrostis stolonifera* L. (Ma et al., 2017). Transcriptome studies performed for pearl millet have mostly covered drought and/or salinity stresses (Dudhate et al., 2018; Jaiswal et al., 2018; Shinde et al., 2018; Shivhare et al., 2020) and only two studies (Sun et al., 2020, 2021) have covered transcriptome of pearl millet under heat stress. Wherein they used only one variety to study the transcriptome, different parts of the plant viz*.* roots, leaves, and whole plant were used for RNA extraction. Given the limited number of studies available on transcriptome under heat stress in pearl millet, it would be appropriate to study the transcriptome of this crop under heat stress to gain novel insights into the underlying mechanism. For the said purpose, we chose two varieties having contrasting feature visa-a-vis their heat stress tolerance (heat tolerant genotype-841-B and heat sensitive genotype PPMI-69).

Flag leaf acts as an immediate source for panicle development during the reproductive stage in plants (Wang et al., 2011), RNA-sequencing of flag leaf subjected to heat stress during flowering stage was carried out using Illumina sequencing platform. *De novo* assembly resulted in 47,310 unigenes and further, functional annotation (gene ontology, corresponding metabolic pathways) was carried out. The aim of the present study was to unravel the gene pool responsible for conferring heat tolerance to pearl millet. Being one among a few transcriptome reports of pearl millet in response to heat stress, the data presented here will be a primary source of information for the research on genomics and functional genomics in this orphan crop.

## Materials and Methods

### Plant Materials, Heat Stress Treatment, RNA Isolation, and cDNA Library Construction

Seeds of two contrasting genotypes, 841-B and PPMI-69, were collected from Division of Genetics, Indian Agricultural Research Institute (ICAR-IARI), New Delhi, India. The genotype 841-B has higher tolerance to heat stress compared to PPMI-69 (Sankar et al., 2013, 2014). The tolerance of the genotypes was evaluated by membrane stability index (MSI) and Malondialdehyde assay (Heath and Packer, 1968). For heat stress treatment, seeds of both the genotypes were surface-sterilized and sown in plastic pots (10 inches) filled with vermiculite and grown under glasshouse (temperature 32 ± 2°C, relative humidity 70–80%, under day length of 12 h), at National Phytotron facility, IARI, New Delhi. 10 seeds per genotype were grown with one seedling per pot. At flowering stage (55 days after sowing), heat stress was applied in a growth chamber at a temperature of 42°C, relative humidity of 70–80%, and normal light conditions for different time intervals (30 min and 6 h). Plants grown at 32–34°C under normal light conditions in the glasshouse served as control. Different plant samples used in the study were given independent identity numbers (Supplementary Table S1). For RNA extraction, one flag leaf per plant was collected from each plant sample (both untreated and treated) respectively in biological triplicates and was immediately frozen in liquid nitrogen before storing at −80°C. RNA isolation was carried out in three biological replicates using TRIzol reagent (ThermoFisher Scientific, United States) and purified using NucleoTrap mRNA mini kit (Macherey-Nagel, Germany). DNA contamination was removed using TURBO DNase (Ambion, United States) according to the manufacturer’s instructions. The RNA quality was assessed using the 2,100 Bioanalyzer (Agilent Technology, United States). One µg of the total RNA from each sample was used to purify poly-A containing mRNA molecules using Oligotex mRNA mini kit (Qiagen, Germany) as described by the manufacturer. Four independent RNA-seq libraries were constructed using TruSeq^®^ Stranded mRNA Library Prep Kit (Illumina, United States) according to the manufacturer’s instructions (Supplementary Table S1). The RNA libraries thus constructed were sequenced using Illumina Hiseq platform.

### Determination of Physio-Biochemical Characteristics of Plants

Malondialdehyde (MDA) content of both the genotypes was estimated as described previously (Heath and Packer, 1968). Briefly, 0.5 g of leaf tissue from each genotype was taken and homogenized in 10% trichloroacetic acid (TCA) and 0.65% thiobarbituric acid (TBA). The homogenate was incubated at 95°C for 30 min and allowed to cool to room temperature (∼25°C) followed by centrifugation at 10,000 × *g* for 10 min. The supernatant was collected and its absorbance was measured spectrophotometrically (Shimadzu UV–vis Spectrophotometer UV-1800, Japan) at 532 and 600 nm. The MDA equivalent was calculated in nMg^−1^ fresh weight as MDA = [(A_532_-A_600_)]/155] × 100. For the estimation of membrane stability index (MSI), the leaf samples were washed with double distilled water (DDW) to remove surface contamination and 10 leaf discs were taken in sealed vials containing 10 ml of DDW separately, followed by incubation at 4°C for 24 h. The electrical conductivity (EC1) was recorded by using a digital conductivity meter (Blum and Ebercon, 1981). For the electrical conductivity (EC2), the samples were autoclaved at 121°C for 20 min, and allowed to cool down to room temperature. The membrane stability index was calculated as per the equation: MSI (%) = 1-(EC1/EC2) × 100.

### *De Novo* Assembly of Flag Leaf Transcriptome

The next-generation sequencing run for whole transcriptome was performed using Paired end (PE) 2 bp × 150 bp library on Illumina HiSeq 2,500. Using FastQC tools (Andrews, 2014), quality check was performed for the raw data shown in Supplementary Table S2. Trimmomatic was used for preprocessing the raw reads to eliminate adapter sequences and poor quality reads. Trinity program was used for *de novo* assembly with the default parameters (Grabherr et al., 2011; Haas et al., 2014). The Cluster Database at High Identity with Tolerance (CD-HIT) program (Li and Godzik, 2006) was run to remove the similar short sequences based on 90% alignment coverage to longer sequence and produces a set of ‘nonredundant’ (nr) representative sequences and eliminating short redundant sequences. The sequences were clustered using TGICL tools (Pertea et al., 2003) with the default parameters to produce longer, more complete consensus sequences. Gene construction was carried out using EvidentialGene tools with the default parameters to retain the biologically significant transcripts.

### Annotation of Transcriptome and Identification of Simple Sequence Repeats and Single-Nucleotide Polymorphisms

The transcriptome structural annotation was performed using TransDecoder (https://github.com/TransDecoder). The functional annotation was performed using BLAST + tools, with BLASTx using a translated nucleotide query (unigenes). Gene Ontology mapping was performed using Blast2GO (Conesa and Gotz, 2008), to specify all the annotated unigenes to various categories such as biological processes, molecular functions, and cellular components. Pathway mapping of unigenes was performed using KEGG database (Ogata et al., 1999). The unigene sequences were aligned to the Clusters of Orthologous Groups (COGs database) (Tatusov et al., 2000) to predict and classify proteins. PlantTFcat online tool (http://plantgrn.noble.org/PlantTFcat/) was used to identify the transcription factor in the generated data. SSRs were identified using MIcroSAtellite (MISA) identification tool. The Microsatellite search module (MISA) is available online for the public (http://pgrc.ipk-ga-tersleben.de/misa/). The SNPs were identified by using GATK best practice pipeline Version 4.1.2.0 (https://software.broadinstitute.org/gatk/best-practices/), the cleaned reads were mapped against the Transfuse. fasta file using BWA aligner (http://bio-bwa.sourceforge.net/). The alignment was performed in default mode. Picard tool was used to co-ordinate, sort, and remove duplicates from the aligned bam files. The GATK tool was used for processing the alignments and variant calling. SplitNCigarReads and HaplotypeCaller from GATK tools were used for reassigning mapping qualities and variant calling, respectively.

### Expression Analysis

Fragments per kilobase of transcript per million mapped reads (FPKM) unit was used to calculate the expression level of unigenes. Read count for each unigenes were calculated and then converted to FPKM using the formula: (Read count X 10^9^)/(Sum of read count × Length). Differential gene expression was determined using DEGseq (Wang et al., 2010), in R package. The significant DEGs were filtered based on the adjusted *p*-value <0.005 and log ratio >1 and −1 between the samples. Heatmaps of the significant genes were generated with the heatmap package (Perry, 2018), in R package. Using R package pvclust (Suzuki and Shimodaira 2006), hierarchical clustering was performed with 1,000 bootstrap replications.

### Quantitative RT-PCR

Total RNA was extracted (as described in Section 2.1) from the treated and control flag leaves to study the differential expression patterns under heat stress (42°C for 30 min and 6 h) of the few selected genes. A total of 10 genes (*PgDnaJ, PgGST, PgNAC67, PgTIL, PgEXP, PgHd1, PgLTP, PgUCP1, PgUCP2,* and *PgUCP3*) involved in heat stress response were selected for differential expression analysis, from the generated pearl millet transcriptome data based on the log2 fold change ≥2 (for upregulated transcripts) and adjusted *p*-values. cDNA was synthesized for studying the expression of selected DEGs with the samples of flag leaves originally used for RNA-seq, using SuperScript^®^ III First-Strand Synthesis System (Invitrogen, United States). qRT-PCR was performed using LightCycler^®^ 480 System (Roche, Switzerland) and KAPA SYBR^®^ FAST qPCR Kits (Kapa Biosystems, United States) was used as reaction components. Gene specific primers were designed using PrimerQuest Tool (Integrated DNA Technologies (IDT), United States) (Supplementary Table S3). PCR program was set as: 94°C for 5 min once followed by 40 cycles at 94°C for 10 s, 60°C for 10 s, and 72°C for 10 s. *PgActin* was used as an endogenous control to normalize all the data (Sankar et al., 2021). 2^−ΔΔCt^ method was used to calculate the relative fold change (Livak and Schmittgen 2001) among the treatments. The significance levels were calculated using two-tailed unpaired *t*-test.

## Results and Discussion

### Determination of Physio-Biochemical Properties of *P. glaucum* Genotypes

The detection of higher content of Malondialdehyde and lower membrane stability index in genotype PPMI-69 compared to that of 841-B indicated that PPMI-69 genotype is susceptible to heat stress compared to 841-B (Supplementary Figures S1,S2). The heat susceptibility of PPMI-69 genotype compared to that of 841-B has been previously reported (Sankar et al., 2013, 2014).

### Illumina Sequencing and Raw Data Pre-Processing

Pear millet, despite being an important source of food and fodder in both arid and semi-arid regions of the world, has not been widely explored as a reservoir of heat tolerant genes. There are some genomic and very few transcriptomic studies exploring the genetic potential of pearl millet as a source of heat stress responsive genes. In the year 2016, Berthouly-Salazar et al. (2016) performed RNA sequencing of different pearl millet populations to explore their climatic adaptability. Varshney et al. (2017) reported whole genome sequencing of pearl millet using shortgun and BAC cloning approaches. Sun et al. (2020) performed Pacbio full-length RNA sequencing of pearl millet under heat and drought stress. Sun et al. (2021) reported root transcriptome of pearl millet under heat stress. Huang et al. (2021) studied the transcriptional changes in pearl millet leaves under heat stress. In this study, the whole transcriptome sequencing was performed using Paired end (PE) 2 bp × 150 bp library on Illumina HiSeq 2,500. The sequencing run produced a total raw data of 288, 876, 956 reads, details are given in Supplementary Table S2. After the removal of low quality sequences, ambiguous bases and adapter sequences by Trimmomatic tool (Bolger et al., 2014), a total of 274, 721, 009 high quality clean reads, containing 39, 782, 593,275 nucleotides (nts) were generated having an average length of 150 nt and GC content of 57.17%. The sequencing data has been deposited to NCBI (National Centre for Biotechnology Information), Sequence Read Archive (SRA) database under the accession number SRP151237.

### *De Novo* Assembly of Pearl Millet Flag Leaf Transcriptome

Using the Trinity program based on the de Bruijn graph algorithm, we performed *de novo* transcriptome assemblies using their default K-mer sizes. The analysis generated 147,934 contigs having the mean length of 1,059 nt and N50 length of 1,526 nt (Supplementary Table S4), which is significantly higher than the average length (725 bp, N50 1,014 bp) reported by Varshney et al. (2017) and lesser than average length (3,102 bp, N50 3,302 bp) reported by Sun et al. (2020). In order to reduce the assembled contig numbers, CD-HIT software was used for grouping and estimating similarity and dissimilarity of nucleotide sequences, which resulted in the number of contigs being reduced from 147,934 to 129,893 due to the removal of redundant sequences. TGICL tools were further used to retrieve longer and complete contigs, as a result, 129,893 contigs were processed into 109,001 contigs, with N50 length of 1,649 nt. In order to retain the biologically significant contigs, EvidentialGene tools was used and these contigs were assembled in a nonredundant manner. 47,310 high quality unigenes were generated, with a total length of 59, 323, 119 nt, a mean length 1,254 nt, N50 length of 1,853 nt, and GC content 53.11% (Supplementary Table S4). The use of assembly tools (CD-HIT, TGICL, and EvidentialGene) led to the improvement of N50 values, compared to raw assembly. To find the read usage in the assembly, we aligned all the 274.721 million reads to 47,310 unigenes using Bowtie two software tool (Langmead and Salzberg, 2012), 72.62% of reads were aligned to the assembled transcripts (Supplementary Table S5).

### Structural and Functional Annotation and Classification of *P. glaucum* Transcriptome

**FIGURE 1:**
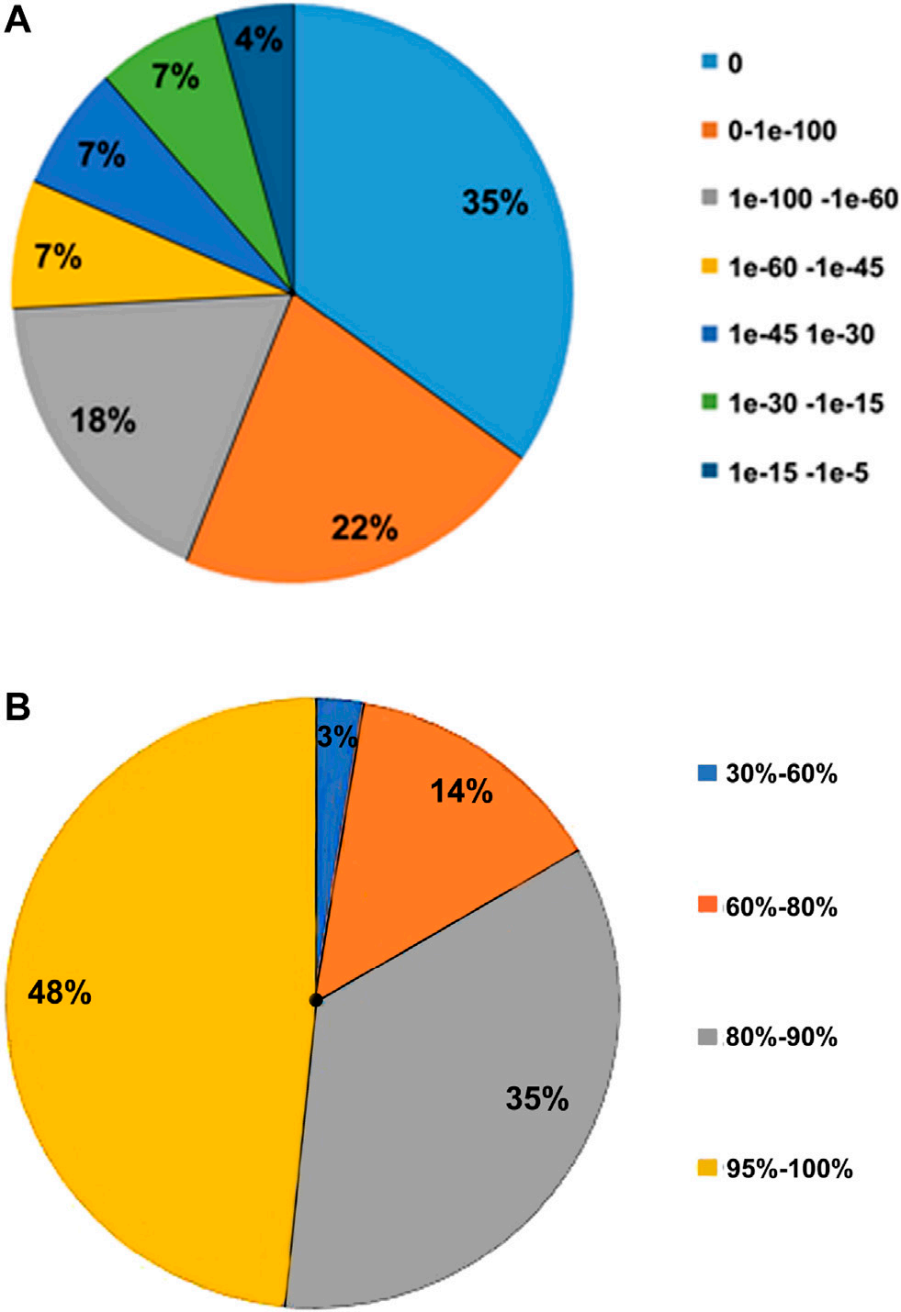
Characteristics of sequence homology of unigenes against the NR database. **(A)**- E-value distribution of BLAST hits for each unigene. **(B)** Similarity distribution of BLAST hits of each unigene.

**FIGURE 2:**
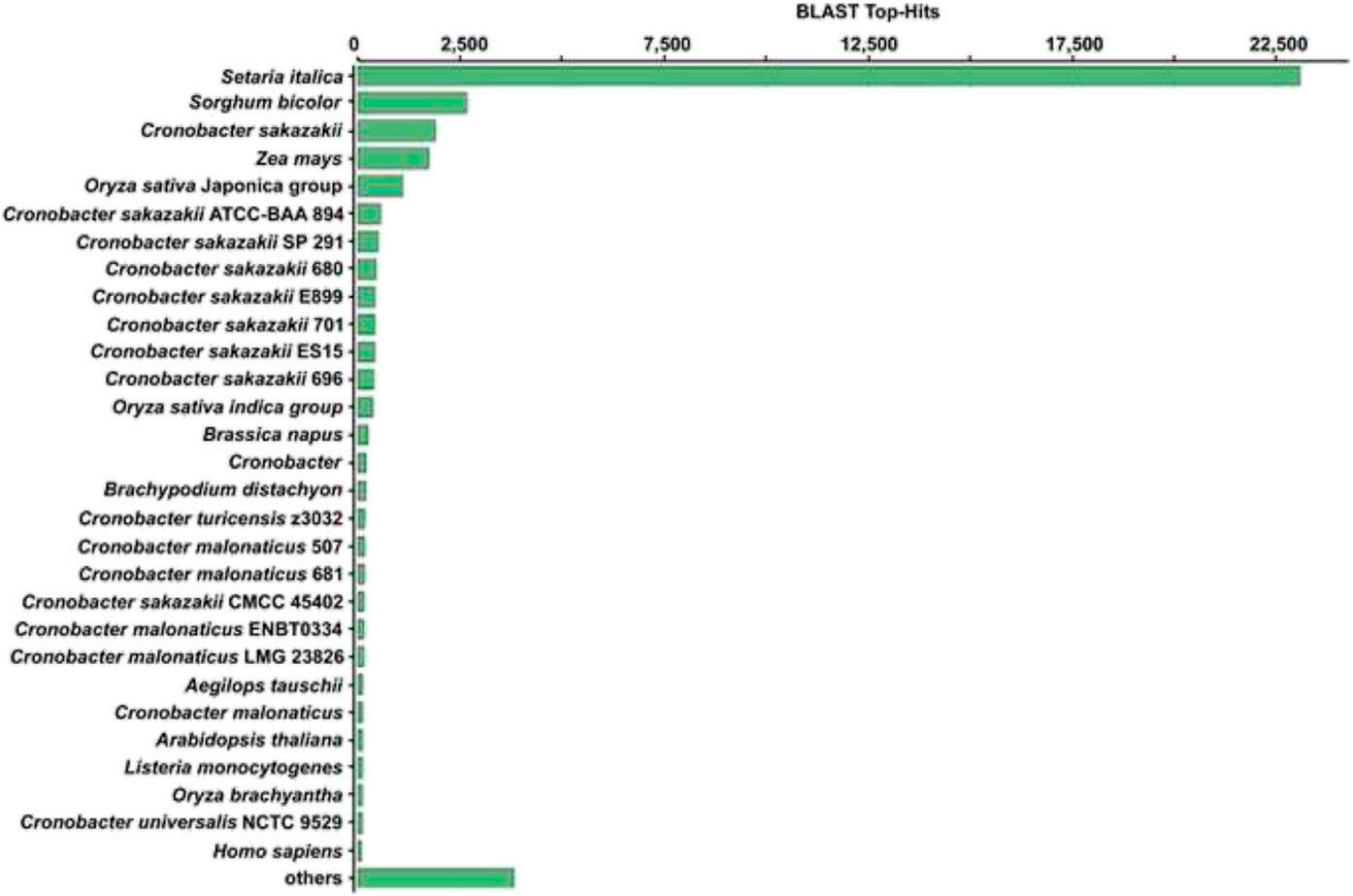
Distribution of the top BLAST hits in different species.

**FIGURE 3:**
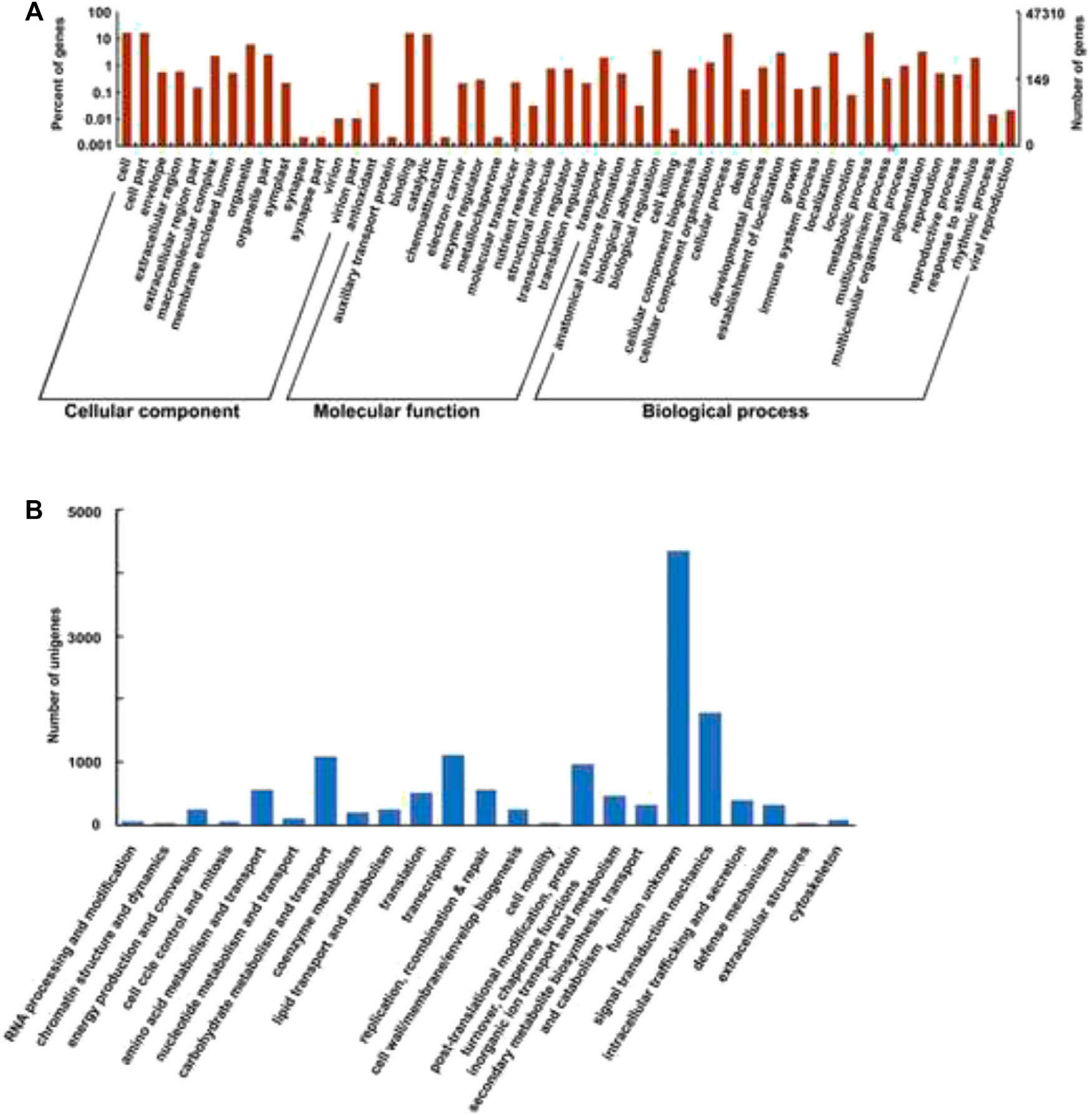
**(A)**- GO (Gene Ontology) classification of the transcriptome. **(B)**- COGs (Clusters of Orthologous Groups) classification.

**FIGURE 4:**
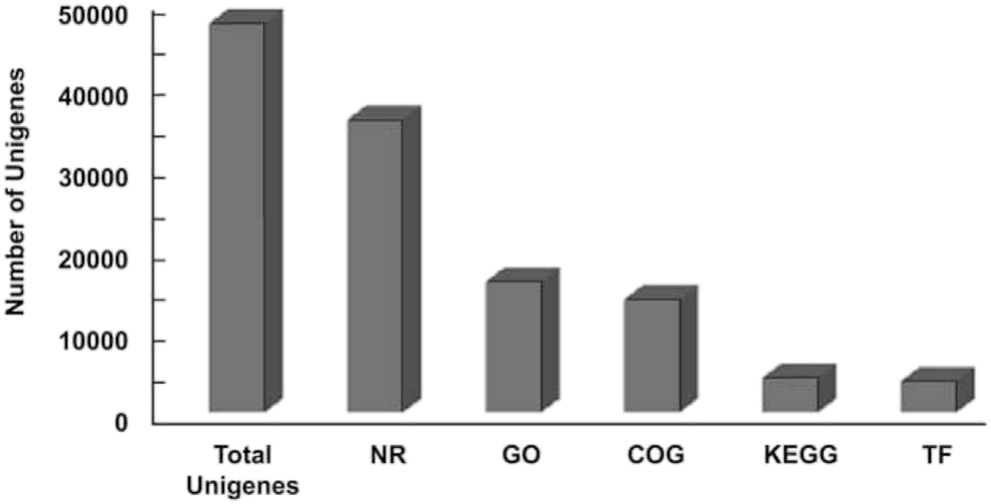
Summary of annotation of all unigenes.

The transcriptome structural annotation analysis was performed using TransDecoder tool (https://github.com/TransDecoder/TransDecoder/wiki-Date of access 17th January 2020). Out of 47,310 unigenes analyzed, 29,919 (63.24%) were found to be coding sequences, in which 11,893 unigenes (25.13%) were detected with ORFs (Open Reading Frame) (Supplementary Table S6). Functional annotation of all the assembled unigenes were compared to the NCBI nonredundant (nr) protein database (Blastx program) (Altschul et al., 1990) with a cut-off value of 1.0 E-06. A total of 35,628 unigenes (75.31% of all unigenes) were annotated against the nr protein database while the remaining 11,682 (24.69%) were not annotated (Table 1). Based on the E-value distribution of Blastx results, 81.34% of the unigenes showed E-value < 1.0E-45 while 18.66% of unigenes had E-value in the range of 1.0E-0.6 to 1.0E-45 (Figure 1A). 83.35% of the aligned unigenes showed more than 80% of similarity distribution (Figure 1B). Blast top hits analysis showed that 57.07% of the annotated sequences correspond to *Setaria italica* (L.) *P. Beauvois*, followed by *Sorghum bicolor* (L.) Moench (6.61%), *Cronobacter sakazakii* (4.72%), *Zea mays* L (4.33%), and *Oryza sativa japonica* (2.74%) (Figure 2). A similar trend in the distribution of BLAST top hits was observed in pearl millet subjected to heat and drought stress previously (Sun et al., 2020). The 30 top-hit species based on nr annotation are shown in Figure 2. Based on Gene Ontology (GO), 15,950 unigenes were designated into three GO classes i.e., 23 biological processes, 14 cellular components, and 14 molecular functions as shown in Figure 3A. Transcriptional sequences for cellular process, biological regulation, establishment of localization, localization, pigmentation, response to stimulus, and metabolic process, among others were significantly enriched under the biological process. Within the cellular component, unigene sequences for the cell, cell part, organelle, organelle part, and macromolecular complex were identified as highly enriched categories. The major proportion of unigenes was assigned to binding, catalytic activity, and transporter categories under molecular function. Moreover, Clusters of Orthologous Groups (COGs) analysis showed that 13,786 unigenes (29.14% of all unigenes) were allocated to 23 COGs categories (Table 1; Figure 3B). Of these, maximum unigenes fall under the category of unknown functions (4,353), followed by a large number of unigenes falling under the categories of signal transduction mechanisms (1,776), transcription (1,105), carbohydrate metabolism and transport (1,095), and post-translational modification, protein turnover, chaperone functions (961). Minimum unigenes were observed to fall under the categories of extracellular structures (9) and cell motility (3). The assigned function of unigenes showed GO (44.77%) and COGs (29.14%) classifications, representing a broad range of cellular transcripts in pearl millet. We used PlantTFcat tool (http://plantgrn.noble.org/PlantTFcat/) and identified 3,841 unigenes associated with the plant transcription factors (Figure 4).

**TABLE 1:** Summary of annotations against publicly available databases.

	Number of unigenes	Percentage (%)
Total unigenes	47,310	100
NR	35,628	75.31
GO	15,950	22.77
COG	13,786	29.14
KEGG	4,263	11.97
TF	3,841	8.119

### Identification of Heat-Responsive Genes Involved in Biological Pathways During Flowering

To identify the potential heat responsive genes and understand their role in various biological pathways, KEGG (Kyoto Encyclopedia of Genes and Genomes) analysis was performed with a cut-off E-value of 1.0E-05. In total, 4,255 unigenes (11.94% of total unigenes) were categorized into 132 KEGG pathways (Table 1; Figure 4). The most represented pathways were the ones related to “biosynthesis of antibiotics” (10.86%), “purine metabolism” (6.46%), “starch and sucrose metabolism” (3.45%), “pyrimidine metabolism” (3.2%), “phenylpropanoid biosynthesis” (2.70%), and “glycolysis/gluconeogenesis” (2.63%). A total of 147 transcripts of starch and sucrose metabolism (3.45%) and 112 transcripts of “glycolysis/gluconeogenesis” (2.63%) were identified. Our study will provide better understanding of molecular mechanisms that are prevailing during the flowering stage of pearl millet under heat stress. Interestingly, this analysis shall help in specifying pathways related to synthesis and turnover of compounds, which have favorable effects in grain filling and yield.

### Identification of Simple Sequence Repeats and Single-Nucleotide Polymorphisms

**FIGURE 5:**
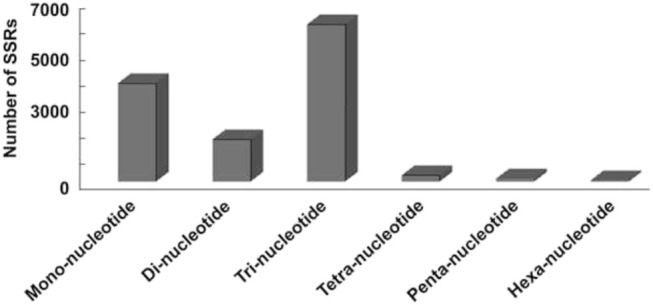
Distribution of SSRs observed in this study. X-axis represents SSRs repeat motif type. Y-axis represents number of SSRs.

Assembled transcriptome of *P. glaucum* was used for the identification of SSRs. Based on the criteria with a minimum of (5–10) repetitions of mono to hexa-nucleotide motifs, MISA software was used to search the SSR markers in all unigenes (Thiel et al., 2003). A total of 12,976 SSRs from 10,294 unigenes were detected, of which 2,116 unigenes had more than one SSR (Table 2). Among all the identified SSRs, 50.88% fall under tri-nucleotide repeats, followed by mono-nucleotides (31.88%) and di-nucleotides (13.7%), but tetra-nucleotide, penta-nucleotide, and hexa-nucleotide were represented only as a small fraction (Figure 5).

**TABLE 2:** Statistics related to SSRs obtained from the transcriptome of *Pennisetum glaucum*.

Total number of unigenes examined	47,310
Total size of examined sequences (bp)	5,93,23,119
Total number of identified SSRs	12,976
Number of unigenes containing SSRs	10,294
Number of unigenes containing more than 1 SSR	2,116
Number of SSRs present in compound formation	986

SSRs are simple motifs of nucleotides (1–10 nucleotides), which may occur as tandem or interspersed repeats and are abundant within the genome of prokaryotes and eukaryotes (Vieira et al., 2016). Genetic variability for heat stress tolerance in *P. glaucum* is still unexplored. Therefore, mining the SSR markers from *P. glaucum* would be utilized by breeders to develop heat stress tolerant crops. Several studies show that SSRs are not distributed randomly along the genome. For example, in case of *Arabidopsis thaliana* (L.) Heynh. rice (Lawson and Zhang, 2006) and *Gossypium raimondii* Ulbr. (Zou et al., 2012), it has been reported that the occurrence of GC-rich trinucleotides SSRs were frequent in exon regions, whereas distribution of AT-rich trinucleotides SSRs were found throughout the genome (coding sequences, untranslated regions, introns, and intergenic spaces) (Temnykh et al., 2001). SSRs are codominant, multi-allele genetic markers that are highly reproducible and transferable among related species (Mason, 2015). As a result, it has been the most widely used marker for genotyping and other breeding purposes (Senthilvel et al., 2008). Identification of new SSRs will provide the necessary impetus to the research community interested in genotyping, genetic mapping, and genetic diversity studies in various *Pennisetum* species (Senthilvel et al., 2008). A total of 305,759 SNPs were observed in the *P. glaucum* transcriptome data. Analysis of SNPs showed that 64.76% (581,800/898,460) of the nucleotide changes were transitions, while 35.24% (316,660/898,460) were transversions. The observed transition: transversion (Ts/Tv) ratio is 1.84. The ratio of transition to transversion is expected to be more than one due to the mutational processes in plant genome. The ratio is lower than the estimates from maize (2.5) and *Arabidopsis* (2.4). In our studies, an average of one variant was observed on every 5,116 bases. The identified SNPs may be utilized as a genomic resource for *P. glaucum* improved by mining alleles of genes and genome assisted breeding for future genome-wide association studies.

### Differential Gene Expression Analysis and Validation of Heat Stress-Responsive Genes

**FIGURE 6:**
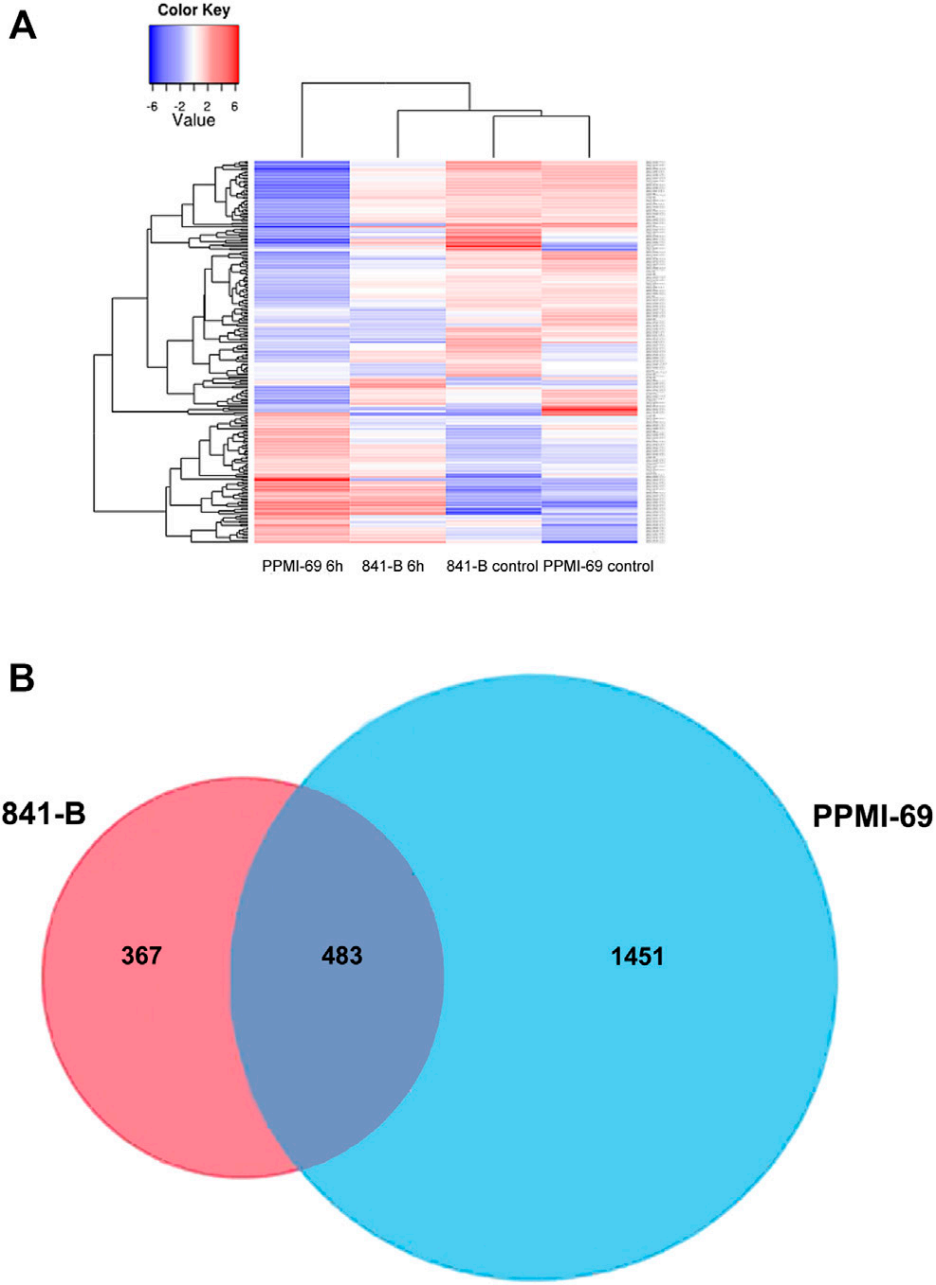
Representation of heat stress responsive genes. **(A)**- Heatmaps of the significant differentially expressed genes with hierarchical clustering. **(B)**- Venn diagram of the differential expressed genes under heat stress conditions in 841-B and PPMI-69 genotypes.

FPKM (Fragments per kilobase per million fragments) unit was used to calculate the expression levels of genes in *P. glaucum* flag leaves transcriptome. Each sample reads were aligned separately to 47,310 unigenes. 75.02% reads got aligned (out of 72, 512, 870) for genotype 841-B control and 68.25% reads got aligned (out of 67, 530, 958) for genotype 841-B 6 h, 72.84% reads got aligned (out of 69, 132, 264) for genotype PPMI-69 control and 74.21% reads got aligned (out of 65, 544, 917) for genotype PPMI-69 6 h. After filtering, based on the adjusted *p*-value, less than 0.005 and (-1< log2 fold change >1) there were 850 DEGs identified between 841-B control and 841-B 6 h, of which 494 genes were upregulated and 356 genes were downregulated. Among 1,934 DEGs identified between PPMI-69 control and PPMI-69 6 h, 964 genes were upregulated and 970 genes were downregulated (Figure 6B).

**FIGURE 7:**
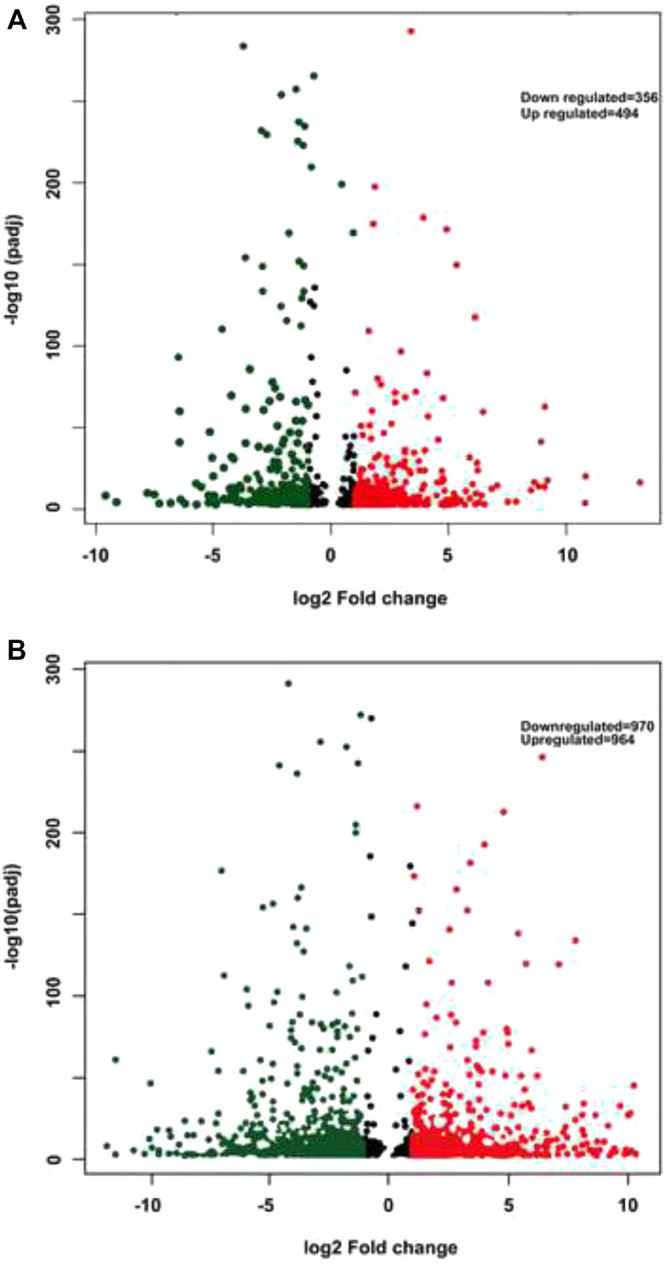
Volcano plots displaying the differentially expressed genes. **(A)**- 841-B genotype. **(B)**- PPMI-69 genotype. Red color represents upregulated genes, green color indicates downregulated genes, while nonsignificant genes are shown as black dots.

Differential gene expression patterns across the treatments and comparison of differential expression patterns between the two different genotypes, were analyzed by cluster analysis with the hierarchical clustering method (Figure 6A) and the DEGs were visualized by volcano plots (Figure 7). The hierarchical cluster analysis of two different genotypes with heat stress treatment shows that the differential gene expression pattern of the transcriptome readily differentiates *P. glaucum* genotype PPMI-69 6 h from others (841-B control, 841-B 6 h, and PPMI-69 control) (Figure 6A), possibly indicating the variation in differential gene expression occurring between the genotypes in response to heat stress. The maximum number of DEGs (1,934 genes) was observed between PPMI-69 6 h and PPMI-69 control (Table 3). These DEGs between the samples 841-B 6 h and PPMI-69 6 h are grouped into two clusters (Figure 6A), representing that the genes involved in thermotolerance have different level of differential gene expression. In general, resemblance in the pattern of DEGs was observed for the samples 841-B 6 h, 841-B control, and PPMI-69. This suggests that 841-B 6 h has better ability to maintain homeostasis during heat stress as compared to PPMI-69 6 h. A comparative analysis between the differentially expressed genes in 841-B and PPMI-69 genotypes (Figure 6B) was performed to identify the common DEGs in both the genotypes under heat stress and those that are unique to each genotype of the *P. glaucum*. A total of 2,301 genes were differentially expressed, 20.99% (483 genes) of which were shared common by both the genotypes. 15.95% (367 genes) of the DEGs were unique to 841-B genotypes and 63.06% (1,451 genes) of the DEGs were unique to PPMI-69. However, the differential expression of genes between the two genotypes cannot be attributed exclusively to the treatments alone, as the two genotypes have inherently different levels of heat stress tolerance and therefore could have different gene expression profile. The sequencing data generated in this study is being utilized for mining and validation of heat stress responsive genes in different varieties of pearl millet including 841-B and PPMI-69 varieties (Sankar et al., 2021).

**TABLE 3:** Statistics related to the number of significantly enriched differentially expressed genes (DEGs).

Comparisons	Up	Down	Total
841-B Control vs. PPMI_69 Control	323	355	678
841-B 6 h vs. 841-B Control	494	356	850
841-B 6 h vs. PPMI-69 Control	813	508	1,321
841-B 6 h vs. PPMI-69 6 h	748	422	1,170
PPMI-69 6 h vs. 841-B Control	970	936	1906
PPMI-69 6 h vs. PPM1-69 Control	964	970	1934

**FIGURE 8:**
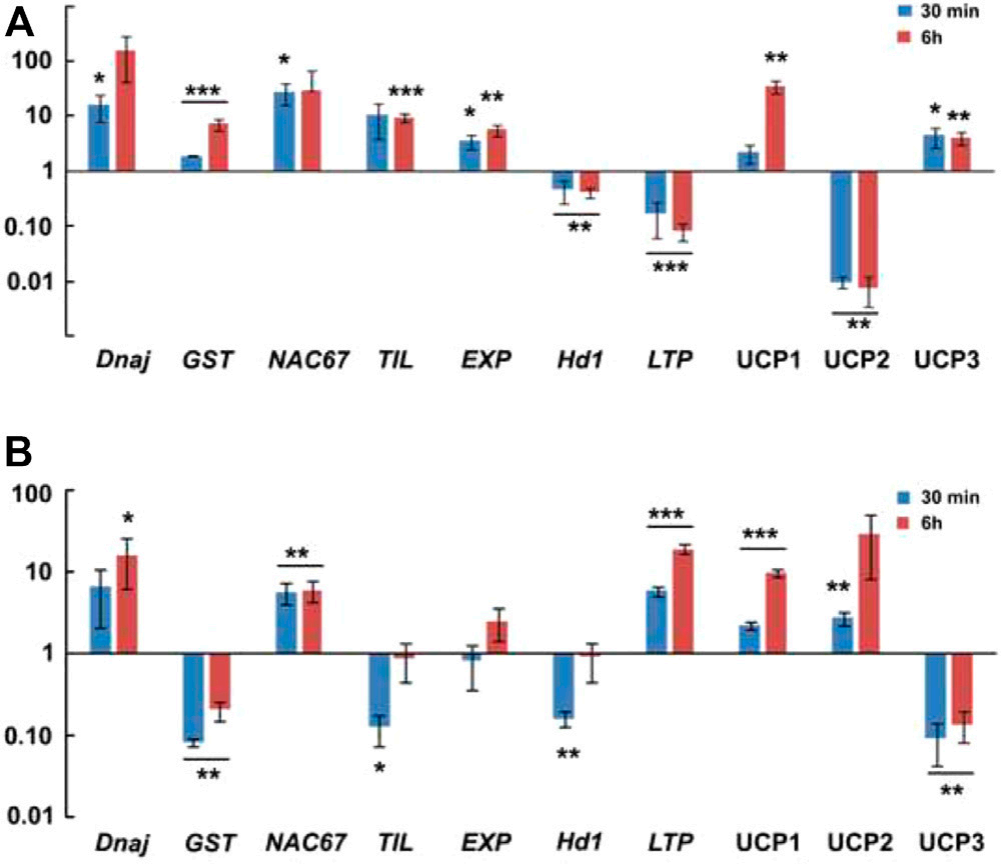
Real time PCR validation of 10 target genes. **(A)**- In genotype 841-B. **(B)**- In genotype PPMI-69. Two tailed unpaired *t*-test was used to calculate *p* value, *p ≤ 0.05, **p ≤ 0.01, ***p ≤ 0.001.

In order to investigate the temporal expression patterns by qRT-PCR analysis, 10 target genes were selected based on the fold changes and adjusted *p*-values. The selected genes displayed varying patterns in response to different durations of heat stress (Figure 8). The validation of selected 10 genes (seven known and three uncharacterized) by qRT-PCR deciphered their significant role in heat stress management in *P. glaucum*. These genes might play essential role(s) in the amelioration of heat stress in *P. glaucum.*

Heat stress induces change in protein conformation and increase improper folding of the native proteins. As a result, accumulation of many heat shock proteins is triggered to counterbalance the negative effect of heat stress in plants (Schlesinger, 1990). Among the many heat shock proteins, DnaJ or Hsp40 is known to play significant role in plant development, signal transduction, and abiotic stresses response, either by itself or in association with Hsp70 (Yang et al., 2010; Shen et al., 2011; Sankar et al., 2021). *PgDnaJ* expression show significant upregulation, about 15 folds in 841-B in response to 30 min heat stress, and 16 folds in PPMI-69 (Figure 8). However, this result indicates the important role of *DnaJ* in maintaining cellular protein homeostasis during heat stress (42°C). Plasma membrane acts as the primary sensor of the cell when the cell is subjected to heat stress (Murata and Los, 1997). Consequently, membrane properties undergo a number of changes in its composition, ion concentration, and ion channels in response to heat stress (Saidi et al., 2010; Prasad, 1996). Peroxidation of lipid membrane is one of the most important change that occurs in the cell in response to various stresses (Thompson et al., 1998). MDA content is a direct indicator of lipid peroxidation. These byproducts of lipid peroxidation, such as electrophiles or xenobiotics, are detoxified by reactive oxygen species (ROS)-scavenging enzymes such glutathione S-transferases (GST), superoxide dismutases, catalases, and ascorbate peroxidases (APX). The expression of *PgGST* in our study was significantly upregulated (7 folds) in response to heat stress in 841-B but significantly downregulated in PPMI-69 (Figure 8), indicating its role in the heat stress tolerance pathway. This result correlates with MDA content (Supplementary Figure S2), as it was observed to be higher in PPMI-69 compared with 841-B. NAC transcription factors (TFs) (NAM, No apical meristem; ATAF, Arabidopsis transcription activation factor and CUC, Cup-shaped cotyledon) play important role in plant growth and development and in regulating response to abiotic or biotic stresses (Souer et al., 1996; Nuruzzaman et al., 2013). Among these NAC genes, *NAC67* has a role in imparting tolerance to multiple abiotic stress such as drought, salt, and cold stresses. *NAC67* has been reported to be involved in conferring tolerance to abiotic stresses in rice (Rahman et al., 2016). However, so far, there has been no report indicating its role in heat stress tolerance. In this study, *PgNAC67* expression was found to be significantly upregulated (27 folds) in 841-B in response to 30 min and six folds in PPMI-69 in response to 6 h of heat stress (Figure 8). It shows that *PgNAC67* plays a role in heat stress response in *P. glaucum*. Temperature-induced lipocalins (TILs), a plasma membrane protein, have an important role in basal and acquired thermotolerance in plant. TILs alleviate the heat induced lipid peroxidation in membrane. *PgTIL* expression was observed to be significantly upregulated (nine folds) in response to 6 h of heat stress in 841-B but significantly downregulated in PPMI-69 (Figure 8). This might be the reason why 841-B is able to maintain a low MDA content (Supplementary Figure S2) as compared to PPMI-69. *PgEXP* expression shows five folds significant upregulation in 841-B in response to 6 h of heat stress (Figure 8). Association of expression genes and heat stress tolerance in some plants has been reported (Xu et al., 2007; Kuluev et al., 2016). Overexpression of the *EXP1* gene exhibits low electrolyte leakage, decrease in membrane lipid peroxidation but higher chlorophyll content, net photosynthetic rate, relative water content, and activity of antioxidant enzyme in transgenic plants (Xu et al., 2014). Heading 1 (*Hd1*) and early heading 1 (*Ehd1*) are mainly known for the regulation of flower development and flowering, leading to either induction or suppression corresponding to the particular photoperiod (Endo-Higashi and Izawa, 2011). Environmental factors such as day length and abiotic and biotic stresses regulate the expression of these genes. Previous studies show that inhibition of early heading 1 (*Ehd1*) in response to drought stress delays flowering in rice (Cho et al., 2017). In our studies, *PgHd1* expression shows significant downregulation in both the genotypes in response to 30 min and 6 h heat stress (Figure 8). *PgLTP* expression shows significant (19 folds) upregulation in response to 6 h of heat stress in PPMI-69 but significantly downregulated in 841-B (Figure 8), indicating the involvement of high activity with regard to transfer of lipid molecules in the cell. This shows active regulation of membrane fluidity in PPMI-69 in response to heat stress. Lipid Transfer Protein (LTP) are reported to be involved with growth and development, response to abiotic and biotic stresses but their functions remain unclear (Debono et al., 2009; Guo et al., 2013; Yu et al., 2013; Pan et al., 2016). Moreover, LTPs has the ability to facilitate the transfer of phospholipids between membranes *in vitro* (Kader 1996). Among the differentially expressed contigs, uncharacterized ORFs share maximum proportion in our study. Uncharacterized genes with a predicted protein domain associated with zinc fingers (ZnF), ribonuclease (RN), and chaperone were validated for their expression during heat stress. Some of the uncharacterized genes were expressed uniquely either in 841-B or PPMI-69. For example, uncharacterized gene *PgUCP1,* with the predicted zinc fingers (ZnF) domain is significantly upregulated (34 folds) in 841-B and (10 folds) in PPMI-69 in response to heat stress (Figure 8). ZnF is known for the involvement in multiple stress response, but their exact molecular mechanism and their interaction is yet to be deciphered (Vij and Tyagi 2008; Bogamuwa and Jang, 2016; Maibam et al., 2019). On the contrary, uncharacterized gene *PgUCP2,* with the predicted ribonuclease (RN) domain is significantly downregulated in 841-B but significantly upregulated (29 folds) in PPMI-69 in response to heat stress (Figure 8). It has been reported that loss of ribonuclease function in *Arabidopsis* enhances its heat stress tolerance (Nguyen et al., 2015). However, uncharacterized gene *PgUCP3,* with the predicted chaperone domain show considerable upregulation (4 folds) in 841-B and is downregulated in PPMI-69 in response to heat stress. These uncharacterized genes could likely represent the important genes involved in imparting variation in thermotolerance among different genotypes. Furthermore, detailed investigation of these uncharacterized genes is required for understanding the role in response to stress.

## Conclusion

This study investigated the transcriptome profile of pearl millet flag leaves in response to heat stress. In this study, high quality 47,310 unigenes were generated and annotated. This data will provide the foundation for research on gene expression, genomics, and functional genomics in pearl millet improvement program. Furthermore, the SSRs obtained in this study shall facilitate the research on genotyping, and diversity studies of this important crop. The candidate genes, whose expression patterns were validated by qRT-PCR, included seven genes known to have role in heat stress in Pennisetum and/or other crops and three uncharacterized genes whose role is yet to be established in heat stress. All these genes along with the pool of differentially expressed DEGs between two genotypes comprise important resource that can be explored for their effective utilization in the development of transgenic crops tolerant to heat stress.

## Data Availability

The datasets presented in this study can be found in online repositories. The names of the repository/repositories and accession number(s) can be found in the article/Supplementary Material.

## Abbreviations

nt, nucleotides; h, hours; BLAST, Basic Local Alignment Search Tool; SSRs, simple sequence repeats; mm, milli metre; ESTs, expressed sequence tags.
