# Supplementary material for: Transcriptome Analysis of Pennisetum glaucum (L.) R. Br. Provides Insight Into Heat Stress Responses
Source: Front Genet. 2022 Jun 2;13:884106. doi: 10.3389/fgene.2022.884106 (PMC9201763; doi:10.3389/fgene.2022.884106)
Supplement: Supplementary file 1 [file DataSheet1.docx]

**Title: Transcriptome analysis of *Pennisetum glaucum* (L.) R. Br. provides insight into heat stress responses**

**Author names and affiliations**

Albert Maibam^a,b^, Showkat Ahmad Lone^bc¶^ Sunil Nigombam^a,b^, Harinder Vishwakarma^b^, Kishor Gaikwad^b^, S. V. Amitha Mithra^b^, Madan Pal Singh^d^, Sumer Pal Singh^e^, Monika Dalal^b^ and Jasdeep Chatrath Padaria^b*^

^a^ PG School,Indian Council of Agricultural Research-Indian Agricultural Research Institute, New Delhi, India

^b^Indian Council of Agricultural Research -National Institute for Plant Biotechnology, New Delhi, India

^¶c^Centre of Research for Development, University of Kashmir, Srinagar, India

^d^Division of Plant Physiology, Indian Council of Agricultural Research -Indian Agricultural Research Institute, New Delhi, India

^e^Division of Genetics, Indian Council of Agricultural Research-Indian Agricultural Research Institute, New Delhi, India

^*^Corresponding author email: jasdeep_kaur64@yahoo.co.in

^¶c^ Present address: Centre of Research for Development, University of Kashmir, Srinagar, India


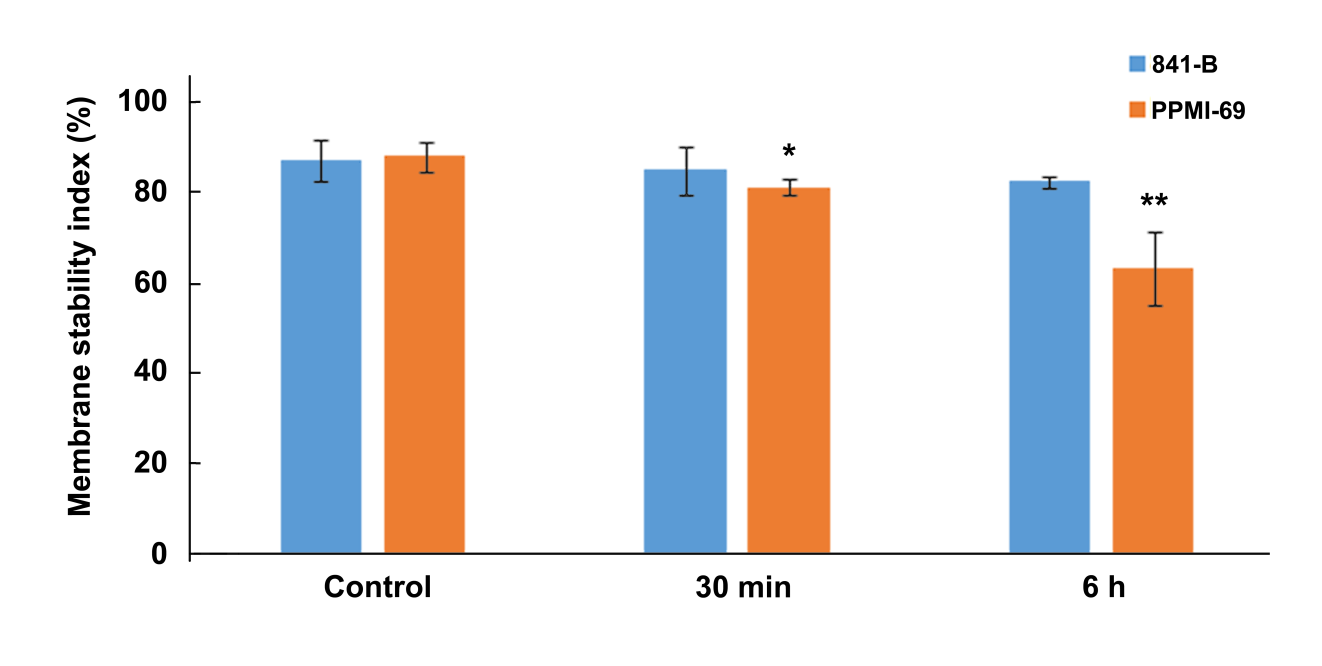


**Supplementary Figure S1**. Membrane stability Index (%) (MSI) under control and heat stress condition imposed at flowering stage in two contrasting genotypes (841B and PPMI69).Two tailed unpaired t-test was used to calculate *p* value, **p*< 0.05, ***p*< 0.01, ****p*< 0.001.


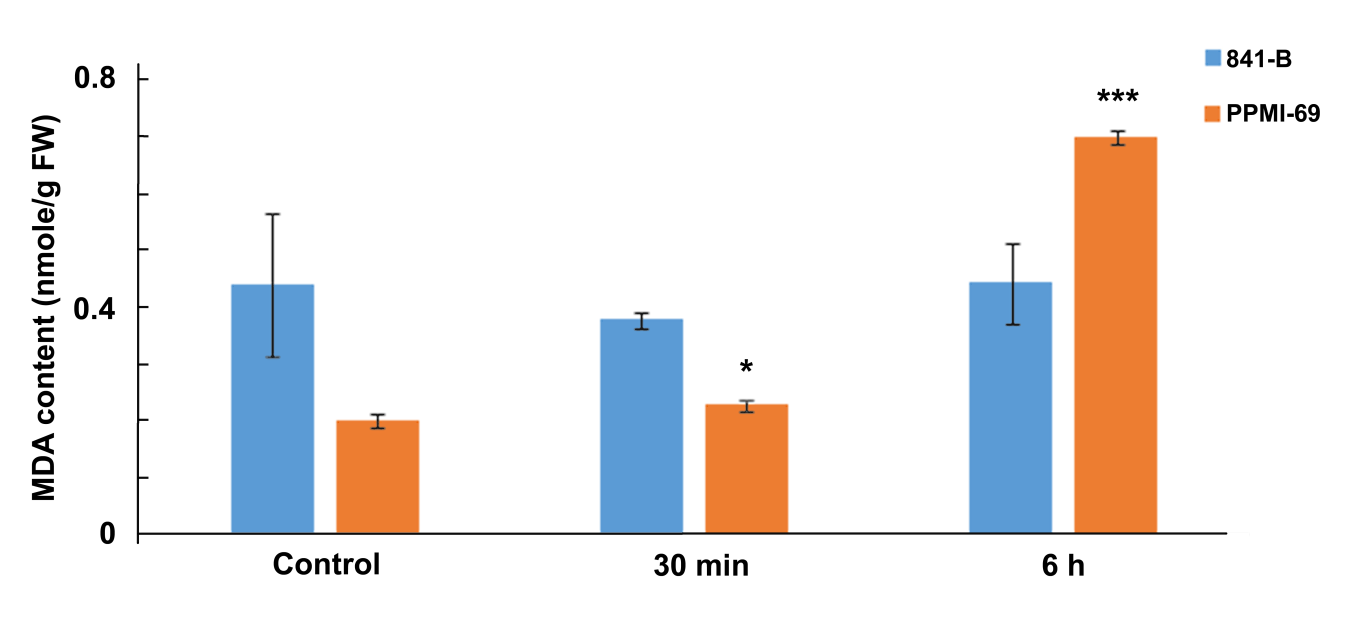


**Supplementary Figure S2**. Malondialdehyde content (n mole/g fresh weight) (MDA) under control and heat stress condition imposed at flowering stage in two contrasting genotypes (841B and PPMI69).Two tailed unpaired t-test was used to calculate *p* value, **p*< 0.05, ***p*< 0.01, ****p*< 0.001.

**Supplementary Table S1. Identification number given to samples used in the study.**

| **Sample ID** | **Genotype** | **Stress conditions** | **Library** |
| --- | --- | --- | --- |
| 841-B | *P. glaucum* genotype 841-B | No stress (control) | 841B C |
| 841-B 30 min | *P. glaucum* genotype 841-B | Heat stress at 42°C for 30 min | 841B HS |
| 841-B 6 h | *P. glaucum* genotype 841-B | Heat stress at 42°C for 6h |  |
| PPMI-69 | *P. glaucum* genotype PPMI -69 | No stress (control) | PPMI-69 C |
| PPMI-69 30 min | *P. glaucum* genotype PPMI -69 | Heat stress at 42°C for 30 min | PPMI-69 HS |
| PPMI-69 6 h | *P. glaucum* genotype PPMI-69 | Heat stress at 42°C for 6h |  |

^Where C stands for control and HS stands for Heat stress^

**Table S2. Summary of RNA-seq data obtained from pearl millet flag leaf**

| **Samples** | **Total raw reads** | **Total clean reads** | **Total clean nucleotides (nt)** | **Total clean nucleotides (nt GB)** | **Average length of clean reads (nt)** | **Q30 %** | **N %** | **GC %** |
| --- | --- | --- | --- | --- | --- | --- | --- | --- |
| 841-B ctrl | 76,413,068 | 72,512,870 | 10,486,909,631 | 10.49 | 150 | 93.04 | 2.49% | 57.80% |
| 841-B 6 h | 71,818,610 | 67,530,958 | 9,724,478,182 | 9.72 | 150 | 92.19 | 2.48% | 56.72% |
| PPMI-69 ctrl | 72,236,940 | 69,132,264 | 10,043,345,563 | 10.04 | 150 | 94.14 | 0.34% | 57.91% |
| PPMI-69 6 h | 68,408,338 | 65,544,917 | 9,527,859,899 | 9.53 | 150 | 94.22 | 0.34% | 56.25% |

**Table S3. Primers used in this study for qRT-PCR analysis**

| **Gene** |  | **Sequence (5′-3′)** |
| --- | --- | --- |
| *PgDnaJ* | F | AATCCATGTTGGACGGGATG |
|  | R | GCTCTTGCGCTGATGTATGA |
| *PgGST* | F | GACTACGAGTACAAGTCGGTAAAT |
|  | R | GAGCGGATGTGCAGGATATT |
| *PgNAC67* | F | TCTCGATTCAGGGAACCAAATAA |
|  | R | CTACTACCGGAACCAGAAACAA |
| *PgTIL* | F | AATACGCGCACCACCTAC |
|  | R | GGAGGTAGAACTTGACCTTGAG |
| *PgEXP* | F | GAGGAGGAGGAGTGAATGAAAC |
|  | R | GTAGCCAGATCGTCGAGTAATG |
| *PgHd1* | F | ACTTGCGCACTTGAGCTATTA |
|  | R | ATGCCTCGTCCACAAACAA |
| *PgLTP* | F | CTACCTACCTACCTCGCTCATA |
|  | R | ACGTGCACATACACATACAGA |
| *PgUCP1* | F | TCCAGTACAGGATGCAAGTTC |
|  | R | CCGAGGACGCGGTTATTT |
| *PgUCP2* | F | GGAGATCCTCTTCTACCCTTTC |
|  | R | GATCCGACGACACACACATA |
| *PgUCP3* | F | TACGAGATGAACCCACCAAAC |
|  | R | CAGGTGAAGCCTAGTTACCATC |
| *PgActin* | F | CCCAAGGCCAATAGAGAGAAG |
|  | R | CACTGGCGTACAAGGAAAGA |

**Table S4. Summary of pearl millet transcriptome assembly**

|  | **Contigs** | **Unigenes** |
| --- | --- | --- |
| Total number | 1,47,934 | 47,310 |
| Total length(nt) | 156,661,397 | 59,323,119 |
| Mean Length(nt) | 1,059 | 1,254 |
| N50 | 1,526 | 1,853 |
| Total consensus sequences | 156,661,397 | 59,323,119 |

**Table S5. Assembly statistics of *P. glaucum* transcriptome**

| **Tools used** | **Trinity** | **CD-HIT-EST** | **TGICL** | **Evidentialgene** |
| --- | --- | --- | --- | --- |
| Number of transcripts | 1,47,934 | 1,29,893 | 1,09,001 | 47,310 |
| Total size of transcripts | 1,56,661,397 | 1,33,060,357 | 1,23,852,445 | 59,323,119 |
| Longest transcript | 14,931 | 14,931 | 23,213 | 23,213 |
| Shortest transcript | 301 | 301 | 301 | 301 |
| Number of transcripts > 300 nt | 1,47,934 | 1,29,893 | 1,09,001 | 47,310 |
| Number of transcripts > 500 nt | 99,692 | 86,416 | 76,452 | 34,992 |
| Number of transcripts > 1K nt | 52,013 | 43,823 | 42,991 | 21,055 |
| Number of transcripts > 10K nt | 19 | 14 | 15 | 11 |
| N50 transcript length | 1,526 | 1,449 | 1,649 | 1,853 |

**Table S6. Structural annotations of pearl millet flag leaf unigenes**

| Total unigene | 47,310 |
| --- | --- |
| Unigene with ORFs | 29,919 |
| Unigene complete ORFs | 11,893 |
| Unigene without ORFs | 5,498 |
